# Advances in Implantable Optogenetic Technology for Cardiovascular Research and Medicine

**DOI:** 10.3389/fphys.2021.720190

**Published:** 2021-10-05

**Authors:** Micah K. Madrid, Jaclyn A. Brennan, Rose T. Yin, Helen S. Knight, Igor R. Efimov

**Affiliations:** Department of Biomedical Engineering, The George Washington University, Washington, DC, United States

**Keywords:** optogenetic, implantable, arrhythmia, wireless, heart

## Abstract

Optogenetic technology provides researchers with spatiotemporally precise tools for stimulation, sensing, and analysis of function in cells, tissues, and organs. These tools can offer low-energy and localized approaches due to the use of the transgenically expressed light gated cation channel Channelrhodopsin-2 (ChR2). While the field began with many neurobiological accomplishments it has also evolved exceptionally well in animal cardiac research, both *in vitro* and *in vivo*. Implantable optical devices are being extensively developed to study particular electrophysiological phenomena with the precise control that optogenetics provides. In this review, we highlight recent advances in novel implantable optogenetic devices and their feasibility in cardiac research. Furthermore, we also emphasize the difficulties in translating this technology toward clinical applications and discuss potential solutions for successful clinical translation.

## Introduction

Optogenetics is a technological approach that utilizes light to control and sense genetically modified neurons or proteins. Though the roots of this technology extend back decades prior, the utility of this concept first broke ground in the field of neuroscience in 2002 when Zemelman et al. developed a method for optically stimulating groups of rhodopsin-sensitized neurons (Zemelman et al., 2002). This light-based methodology gained widespread traction in the field after the seminal publication by Boyden et al. in 2005 (Boyden et al., 2005). Here, cultured rat neurons transfected with the light-gated proton channel Channelrhodopsin-2 (ChR2) uncovered an effective and simple-to-use method for driving neuronal depolarization with blue light. This feat addressed the longstanding need in neuroscience to selectively control and decipher anatomically dispersed groups of neurons, tasks not previously attainable by pharmacological or electrical means (Zemelman et al., 2002; Entcheva, 2013; Joshi et al., 2020). The fast kinetics and cell-type specificity of this new technology led to exponential growth in the field of neuroscience, and in 2010, it was named Nature Methods’ “Method of the Year” (Adamantidis et al., 2015; Boyle et al., 2018). To-date, nearly 8,000 papers have been published on optogenetics. Now, almost 20 years since its original proof-of-concept, optogenetics is making significant headway into cardiovascular research and other areas of medicine.

In cardiovascular research and development, there are three main areas of study in which optogenetics holds unique promise: basic scientific research, optogenetic pacing, and optogenetic termination of arrhythmias. First, in the realm of basic scientific research, the use of light has been a longstanding tool in understanding healthy and abnormal cardiac electrical functioning. In 1976, optical mapping was developed as a means to temporally and spatially measure cardiac action potentials (Salama and Morad, 1976). Optical mapping is now a widely used fluorescent-imaging technique that allows for the visualization of both cardiac action potentials and calcium transients through the use of potentiometric dyes. In optical mapping, light is used as a sensor. On the other hand, in optogenetics, light can be used as a sensor or an actuator. Light as an optogenetic actuator creates myriad opportunities for unprecedented characterizations of electrophysiological properties. For instance, opsins can perturb selective cations within a cell (e.g., H^+^, Na^+^, K^+^, and Ca^2+^) to exclusively stimulate selective inward currents for the examination of individual channel effects in selective areas of the heart (e.g., cardiomyocytes, purkinje cells, or neurons) under a variety of healthy or unhealthy conditions. Furthermore, optogenetics can be combined with optical mapping to allow for “all-optical” cardiac investigations. All-optical approaches provide high spatio-temporal resolution for both sensing and actuating in a purely contactless manner (O’Shea et al., 2019). The excitation wavelength of ChR2 (∼470 nm) works well with properly selected voltage and calcium sensitive dyes; for instance, the voltage-sensitive dye (di-4-ANBDQBS) and calcium sensitive-dye (Rhod-4) have Ex/Em wavelengths at 640/722 and 540/590, respectively, and since the absorption spectra of these dyes do not interfere with one another, or the opsin, the system works. Klimas et al. successfully demonstrated the capacity of such an all-optical system for electrophysiology studies in human stem-cell derived cardiomyocytes (Klimas et al., 2020). Of course, all-optical approaches only provide relative measurements of physiology (e.g., Vm, Ca^2+^), making it difficult to determine absolute values of membrane potentials.

Second, in addition to basic scientific interrogations in the laboratory setting, cardiac optogenetics also has the potential for translational biological control (Entcheva, 2013). Light-based pacing offers the ability to induce cardiac excitability in a clinically relevant manner (Nussinovitch and Gepstein, 2015). Currently, only external pacemakers are available to electrically correct irregular heart rhythms and treat debilitating conditions such as heart failure. These devices restore normal rhythm in the whole heart through brief depolarization of small groups of cardiomyocytes. Though nascent in its clinical translation, optogenetic pacing is a beneficial alternative to traditional electronic pacemakers. Conceptually, the optical stimulus strength needed to trigger a response is lower than that of external electrical stimuli, as it only needs to act on a focused group of opsin-expressing cardiomyocytes rather than a large extracellular space for cellular excitation. Furthermore, the technology itself is more physiologic in nature, as selective stimulation of action potentials via ion channels closely mimics that of native excitable cell activation (Zgierski-Johnston et al., 2020). Additionally, unlike electrical devices, optogenetic stimulation only requires targeted light access, so it does not require invasive surgical procedures or bulky delivery hardware (Joshi et al., 2020).

Finally, optogenetics has the potential to be used as a therapy for the termination of arrhythmias. Electrophysiological function disorders such as ischemic heart diseases, cardiomyopathies, channelopathies, myocarditis, genetic abnormalities, and congenital defects, can result in arrhythmias that have the capacity to lead to sudden cardiac arrest (Joshi et al., 2020). Electronic defibrillators are commonly adopted clinical tools for terminating life-threatening ventricular arrhythmias, but they require depolarization of large areas of cells with high energies. They also can result in non-selective excitation of nerves, muscle damage, discomfort, and even pain from irreversible electrochemical reactions (Joshi et al., 2020). Optogenetic termination of arrhythmias is a particularly appealing alternative technology to cardiac defibrillators because light-based defibrillation negates the need for high energy shocks. Overall, the high spatial and temporal resolution of optogenetics hold great promise for the rapid, precise, and controlled termination of ventricular arrhythmias (Joshi et al., 2020).

The field of cardiac optogenetics has already come a long way, with successes evident in isolated cardiomyocytes, cellular monolayers, Purkinje fibers, neural cells, and whole hearts (Pianca et al., 2017). A number of proteins, cells, or tissues of interest can now be rendered optically sensitive through the delivery of viral-vectors (typically adenoviruses or adeno-associated viruses), enabling either light-based depolarization or light-based hyperpolarization (O’Shea et al., 2019). Since its initial isolation and cloning, ChR2 has also undergone several alterations with application-based modifications (e.g., ReaChR for deeper tissue penetration, CatCh for calcium permeability or ChR2-H134R for enhanced conductance) (O’Shea et al., 2019). Such developments now allow for enhanced selectivity with options for diverse spectral and kinetic properties. However, in order for optogenetic technologies to feasibly make their way into the clinic, there is need for both the efficient delivery of opsins into the area of interest as well as the efficient non-invasive access of light stimulation.

For the excitation of opsins, implantable optogenetic devices hold much promise. In this review, we discuss recent advances in optogenetic technology for implantable studies. Specifically, we highlight papers with unique design elements that can fortify subsequent implantable optogenetic studies, focusing on those that are conformal, transparent, and wireless. We conclude with a discussion of the clinical limitations of these technologies, and we provide insights into future alternatives.

## Overview of Implantable Optogenetic Devices

The use of optical interfaces for investigating cardiac diseases has evolved as a crucial technology for both diagnostics and therapeutics. Implantable systems can measure a broad range of biophysical, chemical, and environmental signals over time for long-term disease screening and treatments. Conventional medical devices succeeded in their targeted therapy but often lacked biocompatibility in the components of their design. The first cardiac pacemaker was designed with a cadmium battery in 1960. Though the initial design never commercially succeeded, it was later improved with different energy sources (Mond and Freitag, 2014). At this time, in order to create ideal therapeutic results, it was difficult to circumvent incompatible design components from cardiac devices. Until limiting components such as the battery, transistors, and overall configuration could be completely controlled, other design elements (e.g., conformal, transparent, and stretchable) could never be developed.

### Flexible and Stretchable Conformal Optoelectronics

Conformal electronics are flexible, stretchable devices that can conform to the diverse topology of specific organ systems to sense and influence physiology. The implementation of this technology in biomedical applications offers important advantages in research, diagnosis, and treatment of disease. However, traditional electronics have been housed on rigid, bittle, and flat substrates. These characteristics are highly incompatible with soft, dynamic organs – such as the heart – and restrict the scenarios in which these devices can be deployed in the body. Conformal electronics solves these limitations by mounting electronic components on deformable substrates in ways that do not disrupt the electronics. In both basic science research and clinical applications, it is ideal that the disruption of native physiology is minimized. These flexible and stretchable electronics are highly suitable for biomedical applications because they allow for soft mechanical coupling to organs to enable physiological sensing and stimulation.

A challenge for conformal devices as a chronic implant is how sufficient power can be supplied to the device. The advent of optogenetics enables optical control of cells and tissue, which is beneficial for conformal electronics because optical stimulation can influence cells with significantly lower power consumption compared to electrical stimulation. Low power consumption means that battery size for powering can be decreased to achieve more miniaturized devices and that wireless devices are increasingly feasible, both of which contribute to implantability. Thus, the optoelectronic configuration is an advantageous next step for conformal electronics.

**FIGURE 1:**
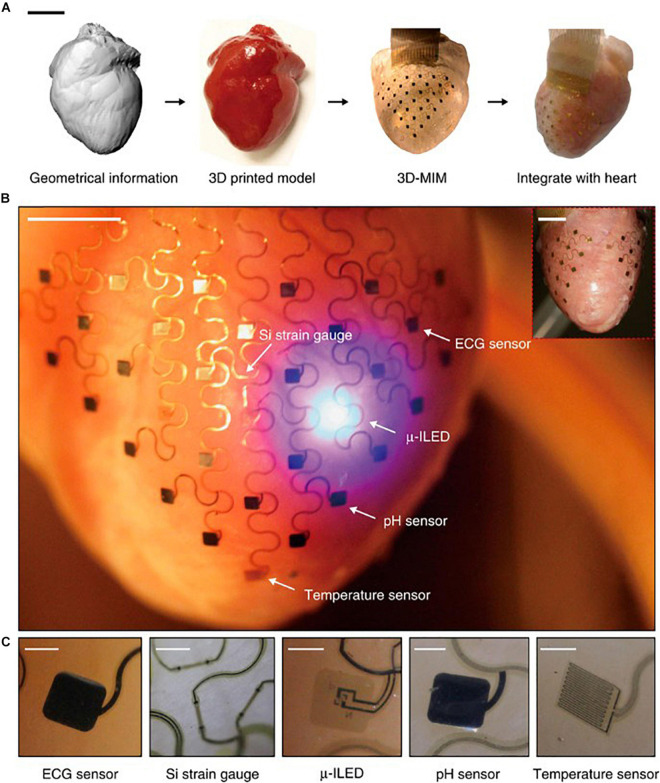
Pericardium-inspired conformal three-dimensional multifunctional integumentary membrane (3D-MIM) for optical stimulation. **(A)** The 3D-MIM substrate is created by capturing the 3D geometry of the heart, rendering a solid model, and casting and curing a thin silicone elastomer. Scale bar, 2 cm. **(B)** A representative image of the 3D-MIM deployed on a Langendorff-perfused rabbit heart with u-ILEDs, pH sensors, and temperature sensors that cover both the anterior and posterior sides of the heart. Scale bar, 6 mm. **(C)** Detailed view of each type of sensor that is integrated into the 3D-MIM. Scale bars, 500 um. Reproduced from Xu et al. (2014).

Conformal optoelectronics can be implemented into already-existing clinical technologies as well as in novel configurations of devices. Kim et al. mounted an array of light-emitting diodes (LEDs) and photodetectors (Kim et al., 2010) onto a balloon catheter (Kim et al., 2011), which is a device commonly employed in non-invasive surgeries. Light-emitting diodes and photodetectors mounted in an array on the balloon retain robust operation despite random bending, folding, and wrinkling during multiple cycles of balloon inflation and deflation. The performance of the electronics does not degrade in the moist dynamic biological environment. The stretchable inflatable nature of the balloon enables soft conformal contact that can be adjusted to accommodate the complex surfaces without disrupting the tissue. Inspired by the native pericardium, Xu et al. developed a novel three-dimensional integumentary membrane that can achieve optical stimulation, pH sensing, mechanical measurements, ECG recording, and temperature sensing across the entire epicardium (Figure 1) (Xu et al., 2014). This elastic membrane was custom-formed to match the geometries of the heart onto which it was deployed. This configuration enables consistent reliable yet non-invasive interfacing to all points on the heart throughout dynamic cycles of the heart. The membrane is outfitted with a variety of sensors and actuators, such as LEDs, electrogram electrodes, pH probes, strain gauges, and temperature sensors. This membrane enables electrophysiological mapping under normal beating conditions where LEDs can provide optical stimulation while sensing electrodes monitor electrical activity. In addition, this membrane can be useful when paired with genetically encoded optical reporters where photodetector arrays can sense intrinsic activity as reported by the genetically encoded indicators to monitor native physiology with minimal disruption. This type of multi-sensing platform presents the future possibility of combined use of μ-LED stimulation and optical sensing by photodetectors for colocalized optical control and recording.

### Transparent Interconnects and Electrodes

**FIGURE 2:**
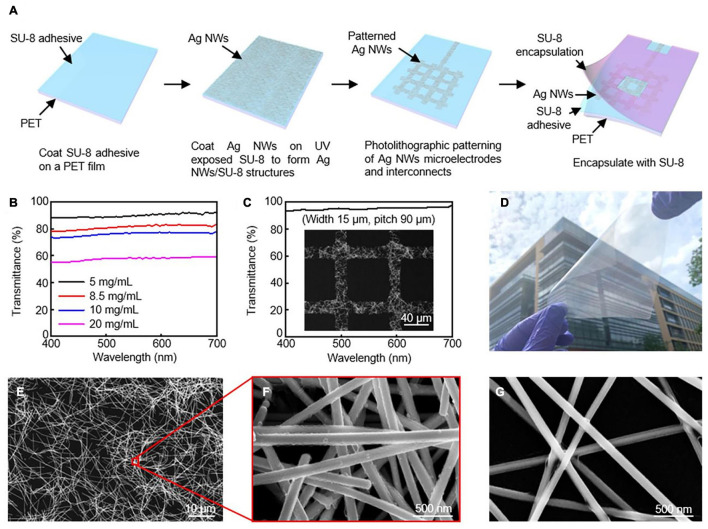
Silver nanowires microelectrodes and interconnects. **(A)** Schematic illustration of fabrication procedure for AgNW microelectrodes and interconnects. **(B)** Transmission spectra for varying concentrations of AgNWs. **(C)** High transmittance AgNW grid from SEM image. **(D)** Optical image of 10 × 10 cm^2^ Ag NW/SU-8/PET film. SEM images of AgNW at **(E,F)** 8.5 mg/mL, and **(G)** 5 mg/mL. Reproduced from Chen et al. (2020).

Transparent interfaces for cardiac research have been developed as a promising tool in optical electrophysiology research (Chen et al., 2020). One-dimensional silver nanowires (Ag NWs) and gold (Au) nanomesh can be tuned in devices to be variably transparent with outstanding electrical conductivity and mechanical flexibility (Figure 2) (Lee et al., 2015 and Seo et al., 2017). Although these tools are highlighted for their transparent properties, Ag NWs and Au nanomesh are also highly flexible and biocompatible components. Other transparent interfaces that have been developed include indium tin oxide, graphene, and carbon nanotubes.

Transparency is directly controlled by the network density of the material in Ag Nanowires. Decreasing the density will in return create open regions of NWs resulting in transparency. This does, however, reduce conductivity of the electrical pathways and effective interfacial regions, reducing electrochemical performance. During the spin coating, the concentration of Ag NWs can be changed to create different levels of transparency. Chen et al. (2020) reports that the average transmittance from concentrations ranging from 20 to 10, 8.5, and 5.0 mg/mLs were: 57.7% to 76.1%, 81.3%, and 90.0% (Figure 2b). Their fabrication strategy can reach high resolutions of approximately 15 μm through photolithography, which is of the highest reported for Ag NWs. Figure 2c shows an image of the grid structure with a transmittance of 95.2%. The fabrication strategy presented in this article was also designed to be able to be upscaled for larger interfaces.

Nanosphere lithography and microfabrication techniques have been used to create Au nanomesh interfaces. With nanosphere lithography, the nanomesh properties can be tuned to control transmittance and sheet resistance by altering properties such as sphere size, metal deposition, and reactive ion etching. Seo et al. (2017) demonstrates electrodes with over 70% transmittance at 550 nm with 8.14 Ω⋅cm^2^. In this study, *in vitro* functionality was validated through cellular testing.

**FIGURE 3:**
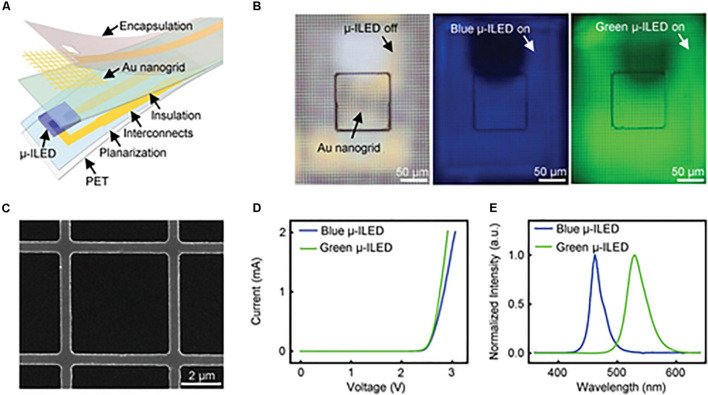
Gold optoelectronic devices for simultaneous optical modulation and electrophysiology possess superior properties including low electrical impedance, high transparency, good cell viability, and superb flexibility. **(A)** Exploded illustration of a multifunctional optoelectronic device containing a transparent gold nanogrid electrode directly on top of a μ-ILED. **(B)** Optical images of a device with μ-ILED turned off (left) and a blue (middle) and green (right) μ-ILED turned on. **(C)** SEM image of a representative Au nanogrid electrode with a width at 500 nm and a line spacing at 5 μm. **(D)** Current and voltage characteristics of the blue and green μ-ILEDs. **(E)** Normalized emission spectra of the blue and green μ-ILEDs. Reproduced from Obaid et al. (2020).

Au nanogrid electrodes can also be colocalized with microscale inorganic light-emitting diodes (μ-ILEDs) for simultaneous electrophysiological recordings and optical actuation (Figure 3). The parameters of this multifunctional system can be easily adjusted to a variety of applications in optogenetics. Obaid et al. (2020) additionally designed this device to be mechanically flexible, highly biocompatible, and to minimize light artifacts during measurement. *Ex vivo* testing showed that they can record abnormal heart rhythms and restore sinus rhythm through optical pacing. Transparent colocalized interfaces are a versatile approach to improving optogenetic research in order to minimize disruption during sensing and stimulation.

### Wireless Devices

Wireless optogenetic devices provide a sophisticated, state-of-the-art means for chronic optogenetic stimulation. Conventional means of long-term or sustained light delivery in genetically engineered animals have relied on lasers or rigid optical fibers held in place with glues, cements, sutures, and external fixtures (Gutruf and Rogers, 2018). However, these physical tethers impose strict limitations on animal movements and lack controlled scalability for *in vivo* studies (Kim et al., 2021). For cardiac optogenetic applications such as chronic pacing or programmed termination of arrhythmias, wireless devices offer unprecedented flexibility as a minimally invasive therapeutic option.

Current wireless technologies are largely either battery-powered or battery-free (Kim et al., 2021). Both technologies offer stable, stand-alone power supplies, but those that are able to function without batteries (such as near-field inductive power transfer or far-field radio-frequency circuits) obviate the need for intermittent battery replacements. Battery-free wireless devices can be fully implanted inside the body, and the control of light delivery for optogenetic control can occur outside the body. Still, there is ongoing work aimed at improving the capabilities of such devices for optogenetics, as present-day wireless technologies require specialized cages with RF power transfers, particular angular orientations, and separate animal studies (as crosstalk can be a concern).

**FIGURE 4:**
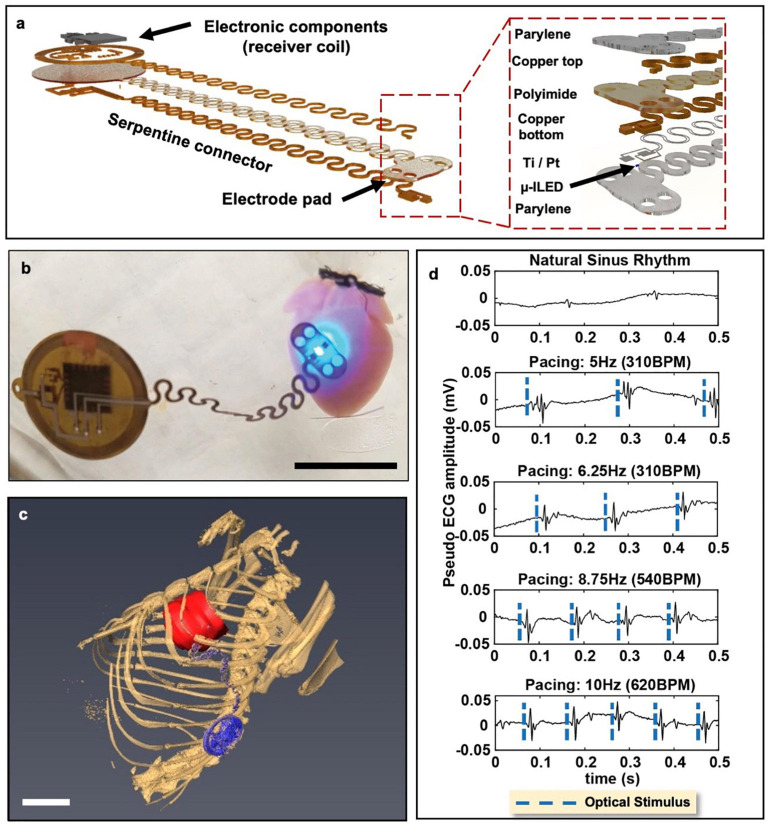
Wireless, battery-free, full implantable optical pacemaker. **(a)** Rendered images of the layered composition of the miniature wireless optical pacemaker. The receiver coil receives energy to power the pacemaker. The electrode pad is placed onto the epicardium for pacing. **(b)** A representative image of the pacemaker performing optical pacing on a ChR2-expressing mouse heart. Scale bar, 1 cm. **(c)** 3D segmentation of anatomical positioning of the pacemaker (blue) with respect to the rat heart (red). Scale bar, 1 cm. **(d)** *Ex vivo* ChR2-expressing mouse hearts were paced at 280 BPM, 310 BPM, 540 BPM, and 600 BPM. Adapted from Gutruf et al. (2019).

An ideal wireless optogenetic system for *in vivo* cardiac pacing is one that is fully implantable, mechanically soft, wirelessly rechargeable, and easily controlled with readily available technologies. Since optogenetics originated for neuroscience, much work to-date has been done with applications focused on the brain or spinal cord (Zhang et al., 2019; Wang et al., 2020). For a thorough review of wireless optogenetic devices with a range of applications, we refer the readers to articles published by Gutruf and Rogers (2018) and Han and Shin (2020). However, it should be noted that many of these technological advancements can similarly be applied to the heart. The first study which created and deployed a battery-free, fully implantable multimodal and multisite pacemaker for applications in small animal heart models was just recently published by Gutruf et al. (2019). Here, a highly miniaturized wireless energy-harvesting device weighing only 110 mg displayed capabilities for subdermal implantation and tolerance to over 200,000 multiaxial cycles of strain without degradation in electrical or optical performance in freely moving ChR2 + rats (Figure 4) (Gutruf et al., 2019).

## Clinical Translation

Cardiac optogenetics could have some benefits over certain pacemaker or defibrillator devices. Ventricular tachycardia for example, can often be treated with pharmaceuticals or regional ablation. However, patients who suffer from ventricular arrhythmias are susceptible to having them recur as well as even sudden cardiac death. These patients often receive implantable defibrillators (ICDs) which deliver strong electrical shocks or antitachycardic pacing to terminate arrhythmias. In order to successfully terminate the arrhythmia, the ICDs need to fill the excitable gap between the trailing edge of the bypassed and leading edge of the reentrant wave (Adgey et al., 2005). Filling the excitable gap with light in optogenetics could be a solution to treating this condition without further pain or damage to the heart. Common methods utilize combined cell and gene approaches to shorten activation times via multisite pacing and resynchronize areas with conduction blocks (Nussinovitch and Gepstein, 2015). Nussinovich also characterizes the use of ChR2 to suppress certain activities of the heart by inducing hyperpolarization. However, the dependability of implantable optogenetic cardiac devices for arrhythmia treatment is limited. Successful device therapy would depend on both stable, safe optogenetic gene delivery and expression as well as a reliable device. In contrast, a totally electrical cardiac arrhythmia therapy would depend only on the functionality of the device. Optogenetic arrhythmia treatment necessitates a “double jeopardy” scenario where the failure modality of the therapy could be either the gene expression or the device itself.

The benefits of optogenetic control are demonstrated in many studies, but the challenge remains to effectively generate opsin expression *in vivo* in a cell-type specific manner. There are a large number of cell specific promoters known that can permit spatially controlled expression to different parts of the heart (e.g., sinus node, atria, ventricle). There are even genetic motifs that could potentially control the expression at a subcellular level to different domains (Koopman et al., 2017). A number of groups have turned to using viral vectors as a means to introduce optogenes into the heart. Adeno-associated viruses (AAV) are recognized to be safe and potentially a target for potential applications in humans (Ferenczi et al., 2019). Adeno-associated viruses vectors have caused a resurgence in gene therapy efforts and there are many ongoing clinical trials. This method has the ability to introduce new genetic material without leaving behind any viral information (Naso et al., 2017). One successful optogenetic study treating ventricular arrhythmias in a large animal model using AAV vectors was performed by Yu et al. (2017). Here, optogenetic modulation of the sympathetic nerves could reversibly inhibit their neural activity to protect against myocardial ischemia-induced ventricular arrhythmias.

Adeno-associated viruses (AAV) gene therapy strategies need to be potent and have high efficacy to achieve long term stable expression for therapeutic intervention. Clinical studies have shown that AAV methods can achieve therapeutic levels in a dose-dependent manner with expression for over a year. Manufacturing AAV vectors is complex and resource-intensive, however, and the optimization of production is an important goal for therapy (Colella et al., 2018). Achieving this would provide a technology that is: (1) capable of efficient transduction, (2) minimally immunogenic, and (3) could trigger persistent expression over time, even without genomic integration (Katz et al., 2017).

Therapeutic optogenetics has received scrutiny for its immunogenic potential with AAV vectors, but there have been numerous methodologies presented to subside many concerns. Such methodologies include removing surface proteins from the virus that cause the immune response and increasing the efficiency and sensitivity of optogenetic proteins in order to reduce the needed strength of expression (Shen et al., 2020). Although currently the technology is immature, by the time therapeutic approaches are ready, these concerns should be addressed.

The effective therapeutic period of optogenetic defibrillation is limited to the length of optogene expression, which is currently approximately one year. On the other hand, ICDs that provide high energy electrical defibrillation can reliably function for five to ten years. Clearly ICDs have a stronger advantage in longer device functional lifetimes, but the high energy shocks that ICDs deliver are physically damaging and psychologically taxing (Mark et al., 2008). The appeal of pain-free optogenetic defibrillation is attractive for improving patient experiences with defibrillation therapy.

## Alternatives to Optogenetics

Due to difficulties in translating optogenetic control into patients, methods for non-invasive optical control are being developed. Graphene has attracted substantial interest due to its unique optoelectronic properties, like high carrier mobility, zero bandgap, and electron hole symmetry. In 2020, Savtcenko et al. present a novel graphene biointerface that does not require the genetic modification of cells but rather capitalizes on the unique optoelectrical properties of reduced graphene oxide (rGO). It is shown that while rGO flakes dispersed at 0.02 to 0.1 mg/ml are in contact with cardiomyocytes, you can optically stimulate the cells at different wavelengths of light. rGO interfaces work through a capacitive energy transfer through electron clouds made at the surface of the polymer (Freitag et al., 2013). This adjacent charge can potentially depolarize whole hearts at the interface between LED devices made for optogenetic tissue. Although this does not currently have the cellular precision that gene therapy can provide, it is a start to a new form of opto-electrical stimulation.

Other facets of control are being developed in transient potential (TRP) channels. TRP channels are a superfamily of cation channels with gates that respond to physical and chemical stimuli (Zheng and Wen, 2019). Thermogenetic tools could be used to drive cardiac activity by increases or decreases in temperature. These tools have been primarily driven through basic science research in the investigation of processes like nutrient uptake, receptor mediated endocytosis and other signaling pathways (Bernstein et al., 2012). X-ray crystallography and Molecular Docking simulations have elucidated critical structural and functional characteristics of these channels (Zheng and Wen, 2019). Although this technology is still very early in its development, there’s potential for integration with cardiac biointerfaces.

## Future Directions

Optoelectronic devices serve as a critical tool to realize the full advantages of optogenetics in monitoring and affecting tissue activity. When paired with genetically encoded fluorescent indicators, photosensors optically monitor physiological activity. Together with transgenically expressed rhodopsin photoreceptors, optical stimulators can modulate biological activity. Implanted optoelectronic devices allow for *in vivo* optogenetic studies in fully conscious freely roaming animals. This configuration enables biological studies in the most physiologically natural environment with minimal hindrance from research instrumentation.

Device-enabled optical stimulation has demonstrated several key advantages, such as lower power requirements and contactless stimulation. Lower power requirements mean that wireless devices and diminished battery sizes are possible. As a result, devices can be further miniaturized and achieve a more non-invasive nature in order to minimally disrupt the body and sense and influence organ systems in a more natural physiological setting.

Transient gene therapy could be possible with bioresorbable devices. These types of temporary devices are composed of biologically benign materials and can self-eliminate *in vivo* via hydrolysis and natural metabolic action in a defined amount of time. Since AAV-mediated gene transfer is effected for up to one year, bioresorbable devices could deliver opsins for local expression and use bioresorbable optical stimulators to administer therapy. Once therapy is complete, both the device and viral transfection self-eliminate in tandem. Although a bioresorbable optical pacemaker for cardiac optogenetic stimulation has not yet been developed, several advancements have been made to this end. Choi et al. demonstrated a bioresorbable cardiac pacemaker that can deliver electrical pacing stimuli for several days and resorb at the end of a therapeutic or study period (Choi et al., 2021). Lu et al. have developed a bioresorbable LED (Lu et al., 2019) that can provide 0.7 mW/cm^2^, which is sufficient for metronomic photodynamic cancer therapy but is not strong enough for optogenetic stimulation of intact ChR2 mouse hearts (Bruegmann et al., 2010). With further innovations in bioresorbable LEDs, a fully resorbable optical cardiac stimulator may be possible.

In considering clinical translation of optogenetics, a major barrier is the requirements for cellular changes at the genetic level so that cells endogenously respond to illumination. Adeno-associated viruses-mediated gene delivery to the heart has been attempted in clinical trials with limited success (Greenberg et al., 2016), although preclinical trials of gene delivery are still ongoing. Whether or not gene delivery to the heart, and therefore clinical optogenetics, can be realized remains to be seen in the coming years. Nevertheless, advances in photoelectric transduction alternatives to optogenetics could still allow us to take advantage of the positive aspects of optical cardiac stimulation. Polymer-silicon nanowire composite meshes (Parameswaran et al., 2018), reduced graphene oxide interfaces (Savchenko et al., 2018), and silicon radial junction stimulators (Liu et al., 2019) can enable photoelectric cardiac pacing without the need for genetic modifications.

Since its emergence nearly two decades ago, optogenetics has served as an important tool in understanding the heart in health and in disease. To further realize the advantages of optogenetic techniques, we must devise new devices and light delivery technologies. Conformal electronics that meld to the curvilinear topology of the heart enable high resolution optical stimulation. Transparent electrodes permit light to penetrate through to achieve simultaneous co-localized electrical sensing upon optical perturbation. The low energy demands of LEDs allow for device miniaturization and wireless tether-free monitoring of cell activity. Currently, the clinical use of optogenetics is fairly limited, but optogenetics and the development of optoelectronic devices has ushered in the possibility of a new optical strategy for pain-free clinical treatment of heart rhythm disorders.

## Author Contributions

MM contributed the abstract, sections related to overview of implantable devices, transparent technology, clinical translation, alternatives to optogenetics, and edits to all sections of the text. JB contributed the introduction and sections conformal and flexible technology sections. RY contributed the sections on wireless device technology and future directions. Each of these authors created the figures in their respective sections. HK performed literature reviews and assisted with the overall formatting and editing. IE devised the scope of the topics, supervised the writing process, and provided feedback. All authors contributed to the article and approved the submitted version.

## Conflict of Interest

The authors declare that the research was conducted in the absence of any commercial or financial relationships that could be construed as a potential conflict of interest.

## Publisher’s Note

All claims expressed in this article are solely those of the authors and do not necessarily represent those of their affiliated organizations, or those of the publisher, the editors and the reviewers. Any product that may be evaluated in this article, or claim that may be made by its manufacturer, is not guaranteed or endorsed by the publisher.
